# Silver Nanoparticles Induce HePG-2 Cells Apoptosis Through ROS-Mediated Signaling Pathways

**DOI:** 10.1186/s11671-016-1419-4

**Published:** 2016-04-14

**Authors:** Bing Zhu, Yinghua Li, Zhengfang Lin, Mingqi Zhao, Tiantian Xu, Changbing Wang, Ning Deng

**Affiliations:** Guangdong Province Key Laboratory of Molecular Immunology and Antibody Engineering, Jinan University, Guangzhou, Guangdong 510632 People’s Republic of China; Virus Laboratory of Guangzhou Women and Children’s Medical Center, Guangzhou, Guangdong 510120 People’s Republic of China

**Keywords:** Silver nanoparticles, Cellular uptake, Reactive oxygen species, Apoptosis

## Abstract

Recently, silver nanoparticles (AgNPs) have been shown to provide a novel approach to overcome tumors, especially those of hepatocarcinoma. However, the anticancer mechanism of silver nanoparticles is unclear. Thus, the purpose of this study was to estimate the effect of AgNPs on proliferation and activation of ROS-mediated signaling pathway on human hepatocellular carcinoma HePG-2 cells. A simple chemical method for preparing AgNPs with superior anticancer activity has been showed in this study. AgNPs were detected by transmission electronic microscopy (TEM) and energy dispersive X-ray (EDX). The size distribution and zeta potential of silver nanoparticles were detected by Zetasizer Nano. The average size of AgNPs (2 nm) observably increased the cellular uptake by endocytosis. AgNPs markedly inhibited the proliferation of HePG-2 cells through induction of apoptosis with caspase-3 activation and PARP cleavage. AgNPs with dose-dependent manner significantly increased the apoptotic cell population (sub-G1). Furthermore, AgNP-induced apoptosis was found dependent on the overproduction of reactive oxygen species (ROS) and affecting of MAPKs and AKT signaling and DNA damage-mediated p53 phosphorylation to advance HePG-2 cells apoptosis. Therefore, our results show that the mechanism of ROS-mediated signaling pathways may provide useful information in AgNP-induced HePG-2 cell apoptosis.

## Background

As one of the most common type of liver cancer, hepatocellular carcinoma (HCC) is the third cause of malignant deaths from cancer-associated worldwide [1–3]. Unfortunately, the diagnosis of HCC is difficult in its earliest stages without screening tests available [4]. Meanwhile, owing to its poor sensitivity to chemotherapeutic agents, high metastatic potential, and resistance to traditional drugs, it is dismal to the overall prognosis of patients with HCC. Thus, it is imperative to develop efficient chemotherapy that has become the great challenge in clinical treatment [5, 6]. In addition, most of current anticancer agents usually have short half-life in the blood circulation and poor aqueous solubility, which hampers therapeutic efficacy of chemotherapy [7, 8]. Recently, nanomaterials have been widely used in biomedical field due to its unique physicochemical properties, including their size distribution, stability of dispersion, morphology, crystalline structure, and thermal properties, that might have the potential to overcome these problems [9]. The increased of application of nanomaterials resulted in raising hopes for employing nanoparticles as alternative anticancer agents [10, 11].

Silver nanoparticles (AgNPs) are less toxic than other form of silver that has obtained an easy access to cells and tissues [12, 13]. AgNPs attract considerable public attention in consumer and medical products compared to other metal materials for their excellent surface enhanced Raman scattering and unique antimicrobial activities [14–17]. Silver nanoparticles have been used widely as antibacterial agents in food storage and environmental [18–21]. Furthermore, AgNPs exhibit low toxicity in humans and have diverse in vitro and in vivo application [22–24]. Moreover, AgNPs have also been shown to combine with corona virus and restrain to bind host cells [25]*.* Thus, exposure to silver nanoparticles as a promising anticancer silver species is becoming increasing intimate and widespread.

Reactive oxygen species (ROS) encompasses highly reactive molecules, including superoxide anion radical, oxygen free radicals, hydroxyl radical, hydrogen peroxide, and singlet oxygen [26]. As a marked imbalance, oxidative stress is the most often described between cellular defense mechanisms and consumption of ROS [27]. The ROS play an important role in many physiological processes. The redox imbalance is associated with many pathologies, such as skin disease, diabetes, cancer, Leigh syndrome, and other diseases [28]. Although many research groups describe the toxicity of AgNP induction of oxidative stress, little is known about the anticancer mechanisms of AgNPs [29]. It is of great interest to ascertain this mechanism of AgNPs. This study was to determine how AgNP-associated changes in redox balance will induce HePG-2 cells apoptosis.

## Methods

### Materials

HePG-2 cells were purchased from American Type Culture Collection (ATCC, Manassas, Virginia). Fetal bovine serum (FBS) and Dulbecco’s modified Eagle’s medium (DMEM) were purchased from Gibco. AKT, p53, PARP, and cleaved caspase-3 antibody were obtained from Cell Signaling Technology. Silver nitrate (AgNO_3_) and vitamin C (VC) were obtained from Sigma. Thiazolyl blue tetrazolium bromide (MTT), 4′6-diamidino-2-phenyindole (DAPI), 2′,7′-dichlorofluorescein diacetate (DCF-DA), bicinchoninic acid (BCA), 6-coumarin, and propidium iodide (PI) were obtained from Sigma. LysoTracker Deep Red was obtained from Invitrogen. The water was supplied by Milli-Q water purification from Millipore in all experiments.

### Synthesis of AgNPs

A stock solution of AgNO_3_ (400 μg/ml) was prepared by dissolving 16 mg AgNO_3_ powder in 40 ml Milli-Q water. Vitamin C (400 μg/ml) was also prepared by dissolving 16 mg vitamin C in 40 ml Milli-Q water. AgNPs were prepared as follows: briefly, 0.1 ml of 400 μg/ml vitamin C was drop by drop added into 4 ml of 400 μg/ml AgNO_3_ under magnetic stirring for 2 h. The excess VC and AgNO_3_ were eliminated by dialysis against Milli-Q water for 24 h. The solution containing nanoparticles was sonicated (KQ-100VDB) by using a water bath for 2 min and pass through filters of 0.2-μm pore size before each experiment. The AgNP concentration was detected by ICP-AES (inductively coupled plasma-atomic emission spectrometry).

### Characterization of AgNPs

The morphology of silver nanoparticles was characterized by transmission electron microscope (TEM, Hitachi, H-7650). The size distribution and zeta potential of AgNPs were detected through Zetasizer Nano ZS (Malvern Instruments Limited) particle analyzer. EDX analysis was carried out on an EX-250 system (Horiba) and employed to examine the elemental composition of AgNPs.

### Cell Viability Assay

HePG-2 cells were incubated in DMEM supplemented with antibiotic and 10 % FBS, penicillin, and streptomycin in a humidified incubator with 5 % CO_2_ atmosphere at 37 °C. The cytotoxicity of AgNPs was detected by MTT assay as previously published [30]. Briefly, cell viability was determined by measuring the ability of cells to transform MTT to a purple formazan dye. HePG-2 cells were seeded in 96-well culture plates at a density of 4 × 10^4^ cells per well at 37 °C for 24 h. Then, the cells were treated with AgNPs at different concentrations for 72 h. After treatment, 20 μl/well of MTT solution (5 mg/ml in PBS) was added and incubated for another 5 h. The medium was removed and replaced with 150 μl/well DMSO to dissolve the formazan crystals. The color intensity of the formazan solution, which reflects the cell viability, was measured at 570 nm using a microplate spectrophotometer (Versammax). The number of test repetitions was three times.

### Intracellular Trafficking of AgNPs

To determine the in vitro cellular uptake of AgNPs, the nanoparticles containing a fluorescent dye 6-coumarin were added to the reaction system. The incorporated dye as a probe for AgNPs offers a sensitive method to determine their intracellular localization. The localization of 6-coumarin-labeled 5 μg/ml AgNPs in HePG-2 cells was monitored by DAPI and Lyso Tracker as before [31]. The treated cells cultured in 2-cm cell culture dishes to 70 % confluence were incubated with Lyso Tracker Red (stained for 2 h), and DAPI H33258 was added (stained for 30 min). The cells were rinsed with PBS three times and then incubated with 6-coumarin-loaded AgNPs from 0 to 120 min which were detected by fluorescence microscope.

### Flow Cytometric Analysis

Flow cytometric was used to characterize the effect of AgNPs on cell cycle distribution as previously reported [32, 33]. Briefly, the cells incubated with AgNPs for 24 h were collected and centrifuged at 1500 rpm for 5 min. The harvested cells were fixed with pre-cooled 70 % ethanol at −20 °C for 24 h. Cells were followed by PI staining for half an hour in the dark. Apoptotic cells with hypodiploid DNA content were measured by using Multicycle software.

### Determination of ROS Generation

The intracellular ROS generation by AgNP-treated HePG-2 cells were detected by staining cells with DCF fluorescence as previously reported [34, 35]. Briefly, cells were collected and suspended in PBS with DCFH-DA at a final concentration of 10 mM at 37 °C for 30 min. Then, the stained cells were collected and resuspended in PBS. The ROS level was examined by detecting the fluorescence intensity using fluorescence microscope and microplate reader with excitation an emission wavelengths set at 500 and 529 nm, respectively. Experiments were performed in triplicate.

### Western Blotting Analysis

The effects of AgNPs on the expression levels of various intracellular proteins in HePG-2 cells were detected by Western blotting as previously reported [36, 37]. HePG-2 cell treatments with AgNPs were incubated with lysis buffer to obtain total cellular proteins. The protein concentration was examined by BCA assay. Protein (10 μg) was subjected to each well. The membranes were incubated with primary and secondary antibodies at 1:1000 dilution in 5 % non-fat milk. Protein bands were visualized with using enhanced chemiluminescence detection reagent (ECL).

### Statistical Analysis

Experiments were performed and repeated at least three times. All the data are presented as mean ± SD. The differences between two groups were analyzed by two-tailed Student’s *t* test. Difference with *P* < 0.05(*) or *P* < 0.01(**) was considered statistically significant. One-way analysis of variance was used in multiple group comparisons. These analyses were carried out using SPSS 13.0 for windows.

## Results and Discussion

### Preparation and Characterization of AgNPs

**Fig. 1:**
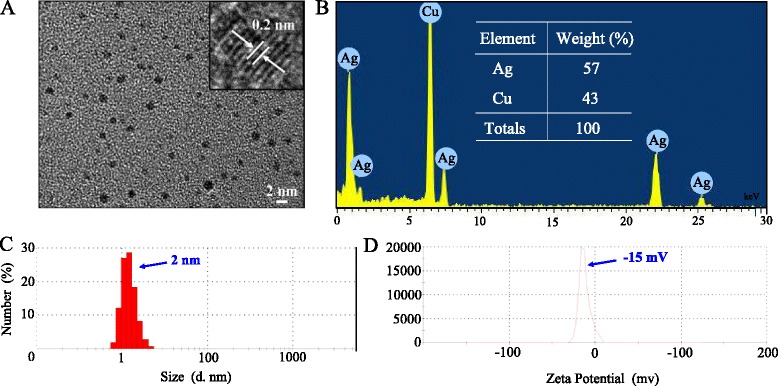
Structural characterization of AgNPs. **a** TEM image of AgNPs. **b** EDX analysis of AgNPs. **c** Size distribution of AgNPs. **d** Zeta potential of AgNPs

In this study, a simple method was used to synthesize AgNPs; various methods were used to characterize the product. As shown in Fig. 1a, TEM images of AgNPs, which demonstrated that the AgNPs presented a uniformity and monodisperse spherical particles, the SAED pattern, and clear lattice fringes (0.2 nm) observed in HRTEM collectively suggested that the nanoparticle possessed a pure crystal structure. In Fig. 1b, EDX indicated the presence of Ag atoms (57 %) with Cu atom signal (43 %). The presence of Cu comes from copper grids. The atom of Ag indicates that silver nanoparticles were successfully prepared. To examine the effects of stability and surface properties of AgNPs, we detected the size distribution and zeta potential of AgNPs. As shown in Fig. 1c, the average particle size of AgNPs was 2 nm. Furthermore, in Fig. 1d, the zeta potential of AgNPs was −15 mV.

### Localization and Uptake Pathways of AgNPs

Endocytosis is one of the primary pathways for cellular uptake of nanoparticles [38]. To examine the intracellular translocation of the nanoparticles, the localization of AgNPs in HePG-2 cells was tracked by simultaneous staining of cell nucleus.

**Fig. 2:**
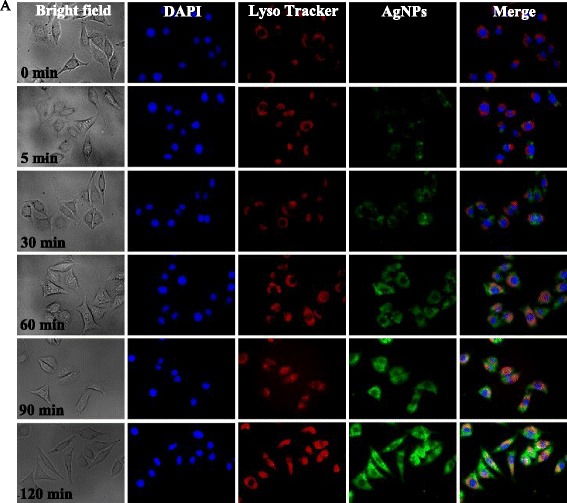
6-coumarin-labeled AgNPs in HePG-2 cells. The HePG-2 cells were treated with 6-coumarin-marked AgNPs for different periods of time and stained with Lyso Tracker Red (lysosome) and DAPI (nucleus) under fluorescent microscope

As shown in Fig. 2, the co-localization of AgNPs and lysosomes was found accumulated in HePG-2 cell membrane and gradually enhanced after then. AgNPs escaped from lysosome after 60 min, transported into the cytosol, and distributed in cells after 120 min. This result demonstrates that lysosome is the target organelle of AgNPs.

### In Vitro Anticancer Activity of AgNPs

**Fig. 3:**
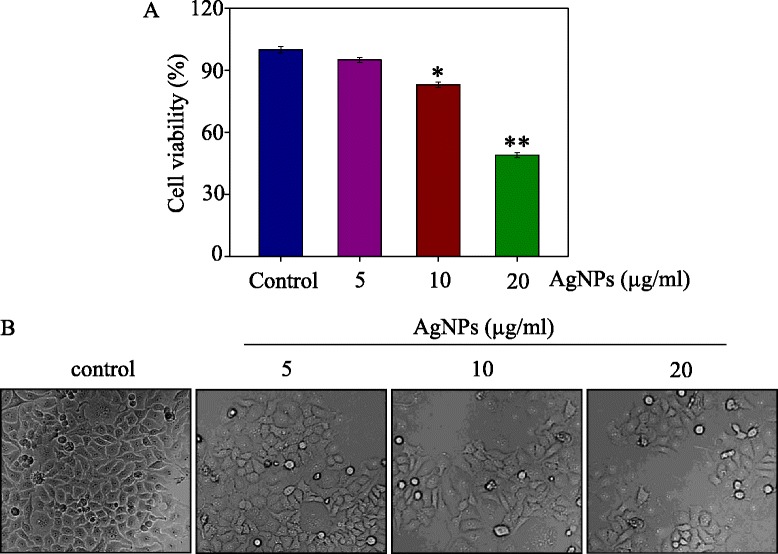
Growth inhibition AgNPs on HePG-2 Cells. **a** Cell viability were treated with different concentrations of AgNPs for 72 h and measured by MTT assay. **b** Morphological changes in HePG-2 cells. Bars with different characters are statistically different at *P* < 0.05(*) or *P* < 0.01(**)

The cytotoxic effect of AgNPs on HePG-2 cells was evaluated by MTT assay. As shown in Fig. 3a, the AgNPs significantly inhibited the growth of HePG-2 cells in a dose-dependent manner.

HePG-2 cells treated with 5 μg/ml AgNPs for 72 h reduced cell viability to 95 %. At the concentrations of 10 and 20 μg/ml, AgNPs decreased the cell viability to 83 and 49 %, respectively. The anticancer activity effects of AgNPs were also further confirmed as shown in Fig. 3b. Cell treated with AgNPs showed cytoplasm shrinkage and loss of cell-to-cell contact. Taken together, these results indicated that AgNPs inhibited cancer cell growth in a dose-dependent manner.

### Induction of Cell Apoptosis by AgNPs

**Fig. 4:**
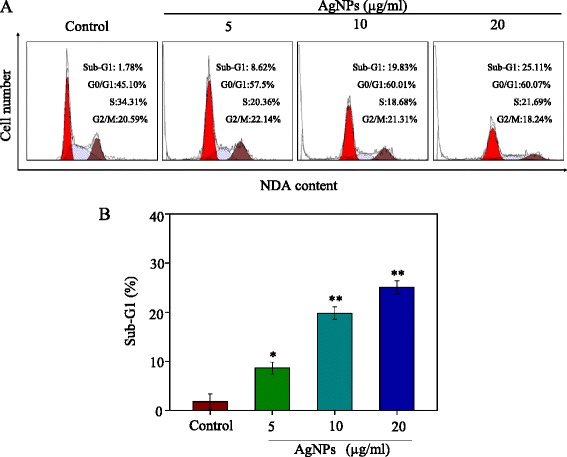
Flow cytometric analysis of HePG-2 cells after treated with AgNPs for 72 h stained with PI after fixation by 70 % ethanol. As shown in **a** and **b**, the cell cycle distribution after different treatments and apoptotic cell population was determined by PI flow cytometric analysis. Bars with different characters are statistically different at *P* < 0.05(*) or *P* < 0.01(**)

Apoptosis plays an essential role in a wide variety of different biological systems, including normal cell cycle, the immune system, embryonic development, morphologic change, and chemical-induced cell death [39]. Therefore, flow cytometry was used to investigate whether apoptosis was involved in cell death by AgNPs. The apoptotic cells which have DNA fragmentation show a typical sub-G1 peak in DNA histogram. As shown in Fig. 4a, b, 5 μg/ml of AgNPs increased the percentage of apoptotic cells to 8.62 %. However, the sub-G1 apoptotic cell population was significantly increased in a dose-dependent manner. At concentrations of 10 and 20 μg/ml, AgNPs increased the apoptotic cells to 19.83 and 25.11 %, respectively. The results indicated that AgNPs depressed HePG-2 cells proliferation.

### Induction of ROS Generation by AgNPs

**Fig. 5:**
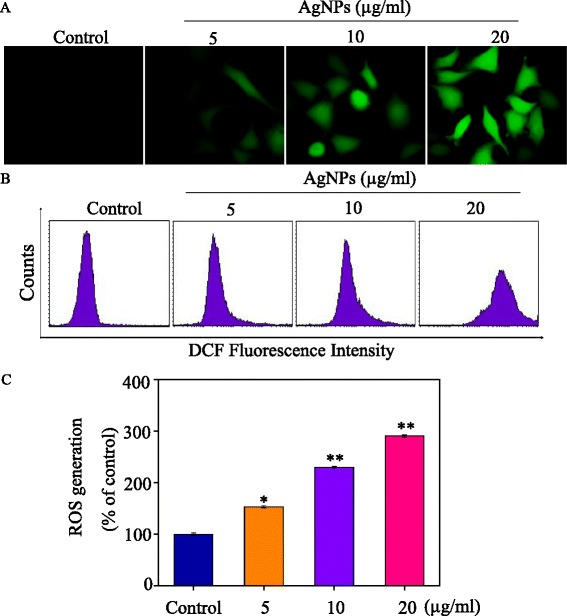
ROS overproduction induced by AgNPs. **a**–**c** Changes of intracellular ROS generation. ROS levels were detected by DCF fluorescence intensity. Bars with different characters are statistically different at *P* < 0.05(*) or *P* < 0.01(**)

ROS has been regarded as a critical activator of cell apoptosis, especially that induced by anticancer drugs [40]. Therefore, in this study, the ROS generation was determined by DCF fluorescence assay to reveal its role the action mechanisms of AgNPs. As shown in Fig. 5a, b, AgNPs gradually increased the intracellular ROS generation in HePG-2 cells; the stronger fluorescent intensity of DCF was found in HePG-2 cells at the concentration 20 μg/ml. As shown in Fig. 5c, AgNPs increased the intracellular ROS generation to 153, 229, and 290 % at concentration of 5, 10, and 20 μg/ml, respectively. The dates indicate that the involvement of ROS may play an important role in the anticancer action.

### Activation of ROS-Mediated Signaling Pathways by AgNPs

**Fig. 6:**
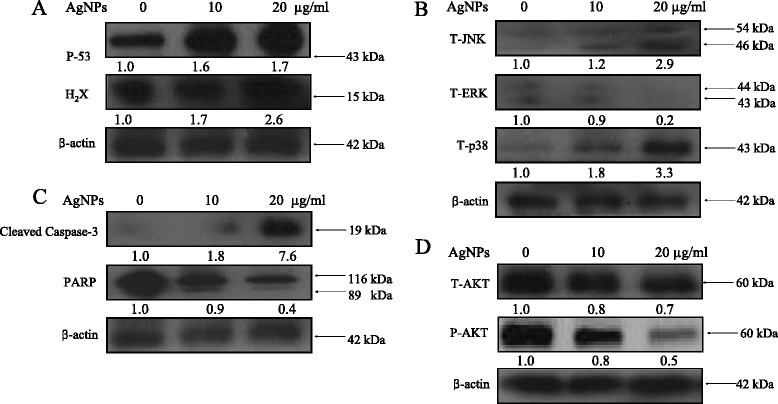
Activation of apoptotic signaling pathways by AgNPs in HePG-2 cells. **a** DNA damage-mediated p53. **b**–**d** The expression level of MAPKs, caspase-3, and AKT in HePG-2 cells. The *numbers* above the images of bands represent the expression level of protein by densitometry analysis

Intracellular ROS overproduction could trigger DNA damage and cell apoptosis by activating of AKT, MAPKs, and p53 signaling pathways. The effects on the ROS-mediated downstream pathways were examined by using Western blotting. As shown in Fig. 6a, treatments of cells with AgNPs significantly increased the expression levels of p53. Meanwhile, as a biochemical marker of DNA damage, H_2_X was significantly activated by AgNPs. Moreover, as shown in Fig. 6b, AgNP treatment triggered differential effects on JNK, ERK, and p38. AgNPs significantly inhibited total ERK and increased the JNK and p38. Meanwhile, treatment of the cells with AgNPs effectively suppressed the expression levels of total AKT in HePG-2 cells as shown in Fig. 6d. The caspase family exhibits important functions in the regulation process of cell apoptosis. Caspase-3 acts as a central regulator in the signaling network [41]. Therefore, the cleavage of caspase-3 and PARP was examined to evaluate their involvement and contribution to cell apoptosis. As shown in Fig. 6c, AgNPs significantly enhanced the activation of caspase-3 and the cleavage of downstream effect PARP. Taken together, these results support that AgNPs induce HePG-2 cells apoptosis by regulation of ROS-mediated AKT, MAPKs, and p53 signaling pathways.

## Conclusions

In summary, a simple chemical method under redox system for preparation of silver nanoparticles has been described in this study. AgNPs exhibit greater abilities to inhibit HePG-2 cells proliferation and enhanced cellular uptake at the average size of 2 nm. AgNPs with dose-dependent manner significantly increased the apoptotic cell population (sub-G1). The studies on the underlying molecular mechanisms revealed that the major mode of cell death induced by AgNPs was cell apoptosis. The mechanisms indicated that AgNPs promote caspase-3-mediated apoptosis by the involvement of ROS generation. Further investigations on apoptotic signaling pathway triggered by the AgNPs in HePG-2 cells are p53, AKT, and MAPKs pathways. Taken together, our findings suggest that AgNP is a prospect silver species with anticancer properties.

## Abbreviations

Ag: silver
AgNO_3_: silver nitrate
AgNPs: silver nanoparticles
DCF-DA: 2',7'-dichlorofluorescein
DMEM: Dulbecco’s modified Eagle’s medium
FBS: fetal bovine serum
MTT: thiazolyl blue tetrazolium bromide
NPs: nanoparticles
PI: propidium iodide
TEM: transmission electron microscopy
VC: vitamin C
